# Papillary thyroid carcinoma with fibromatosis-like stroma: a case report and review of the literature

**DOI:** 10.1186/s12902-023-01337-y

**Published:** 2023-04-14

**Authors:** Antonio Toniato, Marco Brusoni, Marica Mirabella, Luca Pomba, Vasileios Mourmouras, Antonio Scapinello, Enrico Battistella

**Affiliations:** 1 Endocrine Surgery Unit, Veneto Institute of Oncology, IOV-IRCCS, Padua, Italy; 2 Anatomy and Histology Unit, Veneto Institute of Oncology, IOV-IRCCS, Padua, Italy

**Keywords:** Papillary thyroid carcinoma, Nodular fasciitis-like stroma, Fibromatosis-like stroma, Desmoid tumor, β-catenin aberrant staining

## Abstract

**Background:**

Papillary thyroid carcinoma (PTC) is a common neoplasia with multiple variants. One of these extremely rare and poorly described variants is PTC with fibromatosis-like stroma (PTC-FMS), a peculiar entity distinguished by its predominant mesenchymal component. This paper reviews the literature, discusses the diagnostic challenges, and the clinical and surgical implications of this type of tumor which has fewer than 30 cases reported in the literature.

**Case presentation:**

We reported a case of PTC-FMS found in a 41-year-old Italian woman, who came to our Institute with a recent growth in the form of a mass on the neck. Further immunohistochemical examination showed β-catenin aberrant staining both in the nuclei and cytoplasm of the mesenchymal cells. The patient underwent total thyroidectomy and received radioactive iodine (RAI) 2 months after surgery.

**Conclusion:**

Given the possibility of recurrence of PTC-FMS and the ineffectiveness of RAI therapy, complete surgical resection represents the main treatment for this type of tumor. Despite the fact that the specific nature of these lesions has yet to be determined, guidelines for classical PTC should be followed.

## Background

Thyroid carcinoma is the most frequent neoplasia of the endocrine system with various histological subtypes. The papillary thyroid carcinoma (PTC) variant is the most common thyroid malignancy, representing up to 80% of all the well-differentiated forms [1]. The incidence of papillary carcinoma has grown in recent decades in several, including Italy, but overall survival and prognosis remained excellent [2]. Up to date several histological variants of PTC have been described. One of these is PTC with prominent stromal proliferation, characterized by two intermingled components: the minor epithelial component displaying the typical features of conventional PTC and the predominant mesenchymal element resembling nodular fasciitis or fibromatosis, hence the names PTC with fibromatosis-like stroma (PTC-FMS) and PTC with nodular fasciitis-like stroma (PTC-NFS). The great majority of cases described in the literature contain > 80% of mesenchymal cells and < 20% of malignant follicular cells [3, 4].

These two histotypes are synonyms and are discussed in the 5th edition of World Health Organization (WHO) as fibromatosis/ fasciitis-like/ desmoid type stroma [3]. However, some authors consider these two variants to be two distinct entities with different clinical courses [4].

This article we reported the first case of PTC with fibromatosis-like stroma from among more than 20′000 thyroidectomies carried out at our Institution from 1990 to 2021.

## Case presentation

During routine follow-up for multinodular goiter, a 41-year-old Italian woman presented with a recent growth in the form of a mass on the right side of her neck. The patient had no other remarkable personal or familial medical history. Physical examination revealed a solid and partly exophytic nodule in the right lobe of the thyroid, mobile while swallowing and without associated pain, dysphonia, or dysphagia. No lateral cervical or supraclavicular lymphadenopathies were detected. Her laboratory blood tests, including thyroid-stimulating hormone (TSH), serum free thyroxine (fT4) and serum free triiodothyronine (fT3), were normal. Additional examinations, such as thyroglobulin, calcitonin, thyroid peroxidase antibodies (TPOAb), and thyroglobulin antibodies (TgAb), were negative. Further investigations with ultrasonography (US) and computed tomography (CT) confirmed the presence of a hypoechogenic and heterogeneous nodule of 3 cm in the right lobe of the thyroid, protruding between the sternocleidomastoid muscle and the internal carotid artery. Fine-needle aspiration cytology (FNAC) was compatible with a TIR 3B nodule according to the Italian Consensus for the Classification and Reporting of Thyroid Cytology (ICCRTC) [5]. The patient underwent total thyroidectomy with central compartment lymphadenectomy (VI level). The postoperative course was uneventful, and the patient was discharged three days after surgery. The patient received radioactive iodine (RAI) therapy (2.59 GBq) 2 months after surgery.

## Histopathology

**Fig. 1:**
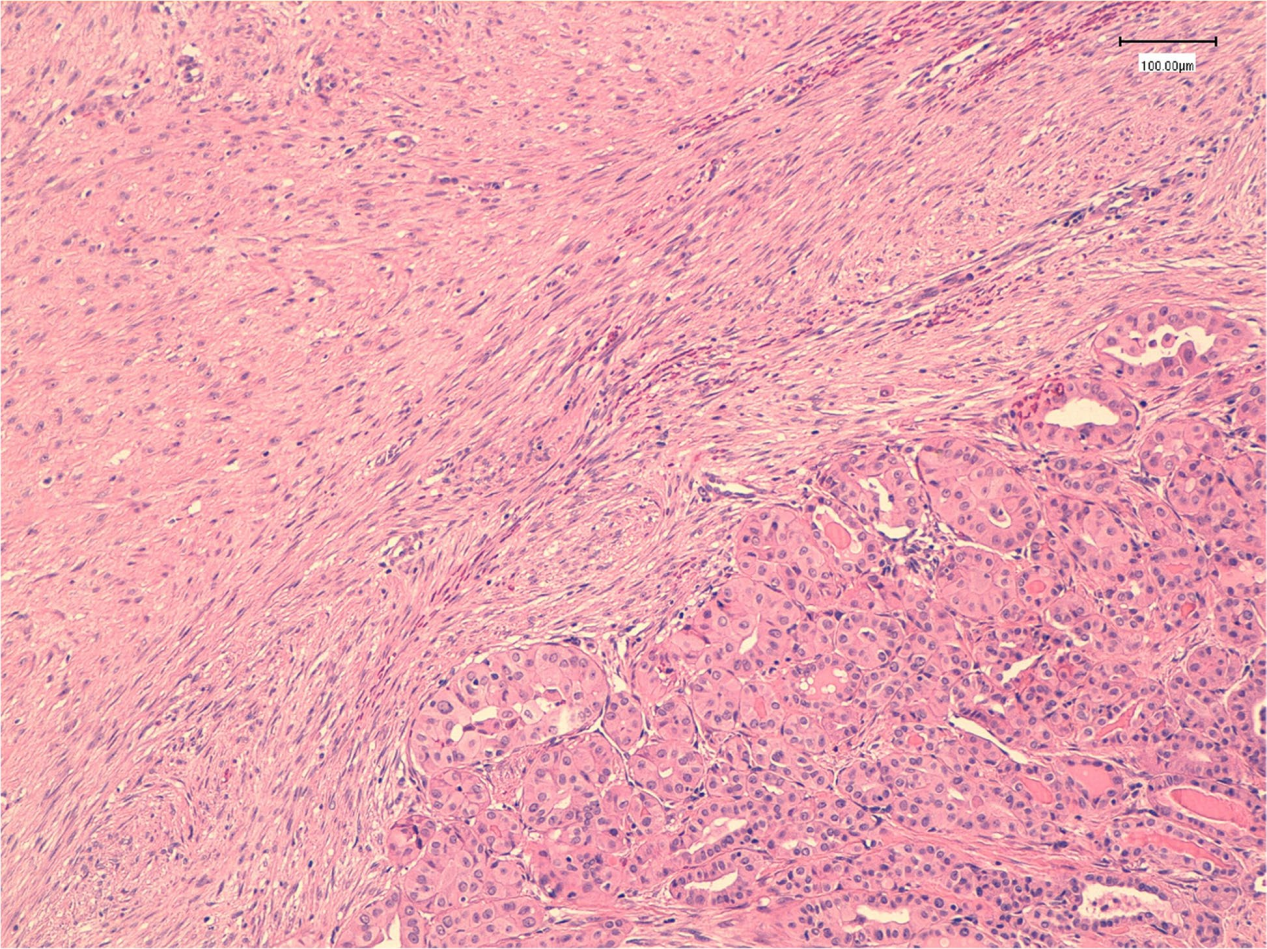
Hematoxylin eosin stain showing two intermingled components of the tumor, the epithelial component (right) with a focus of papillary thyroid carcinoma and the stroma (left) consisting of spindled-shaped cells arranged in fascicles (H&E: original magnification 100x)

At macroscopic examination, the left lobe measured 3.0 × 2.8 × 1.3 cm, the isthmus measured 1.5 cm and the right lobe measured 4.0 × 3.2 × 1.8 cm. The cut sections of the specimen showed a fasciculated and whitish solid nodule of about 2.3 cm with an irregular margin, located between the upper and the medium third of the right lobe and extended to the peri-thyroid soft tissues. Microscopically, the tumor consisted of 2 components. The mesenchymal component, representing 70% of the tumor, was composed of desmoid stroma with fibromatosis/fasciitis-like aspect (Fig. 1). The epithelial component, representing 30% of the tumor, was consistent with papillary thyroid carcinoma. The tumor was staged as pT3b N0 according to the America Joint Commission on Cancer (AJCC 8^th^ ed.).

**Fig. 2:**
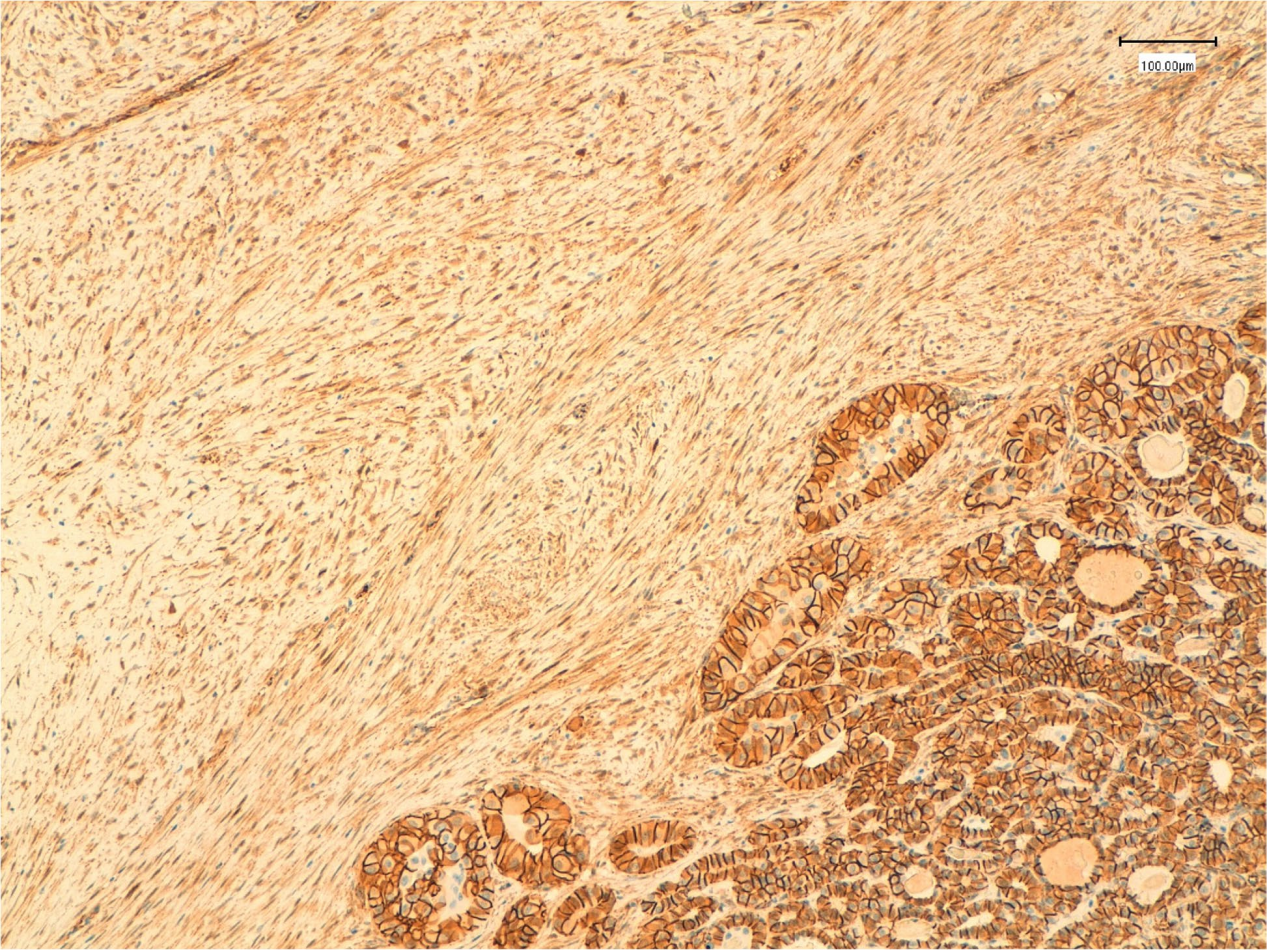
Immunohistochemical staining for beta-catenin showing diffuse nuclear and cytoplasmic reactivity in the stromal component (left), whereas the epithelial component demonstrates diffuse membrane positivity and nuclear negativity (left) (original magnification 100x)

**Fig. 3:**
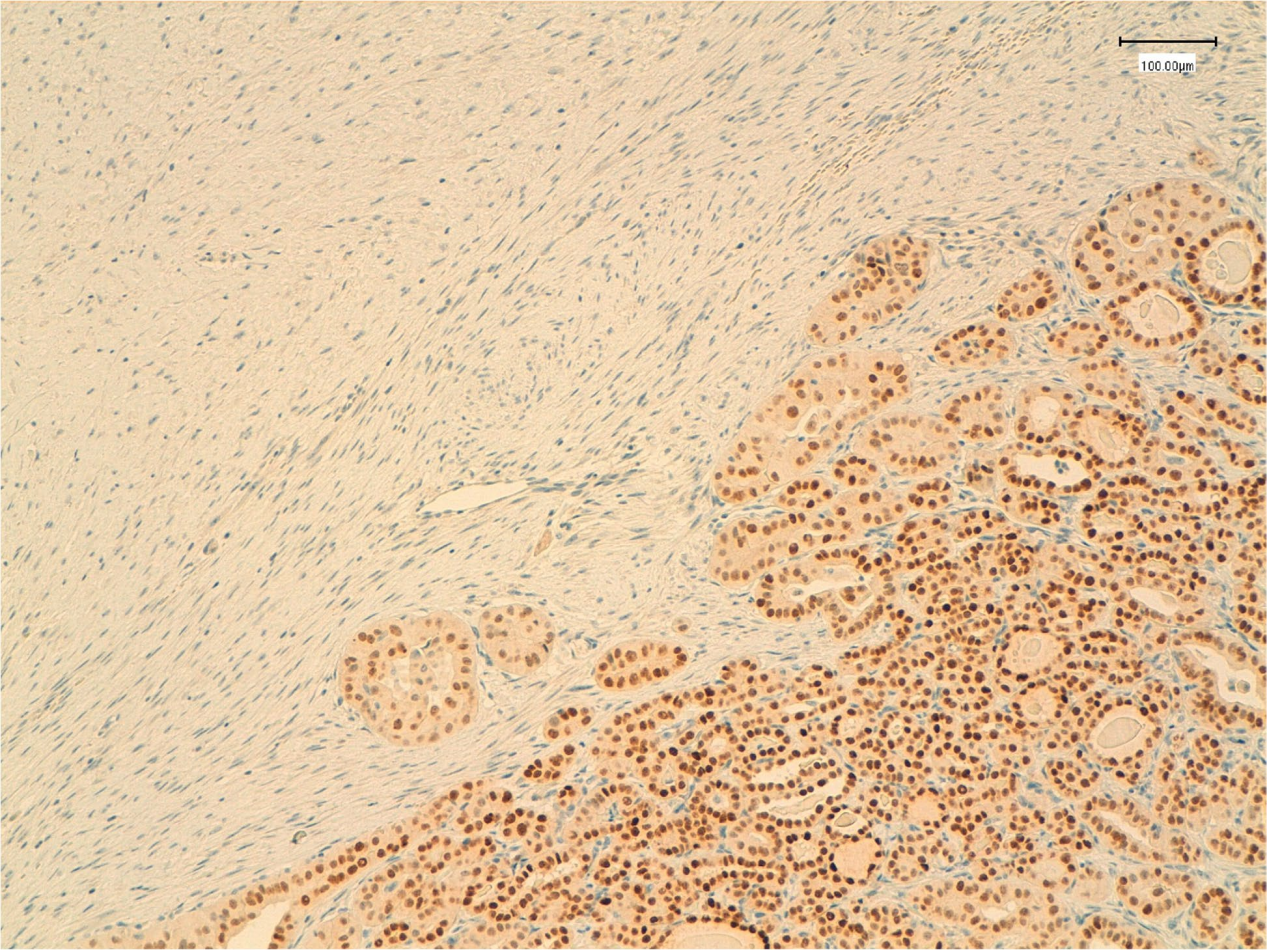
Immunohistochemical staining for TTF1 showing strong nuclear positivity in the epithelial component (right), while the stromal component is negative (left) (original magnification 100x)

**Fig. 4:**
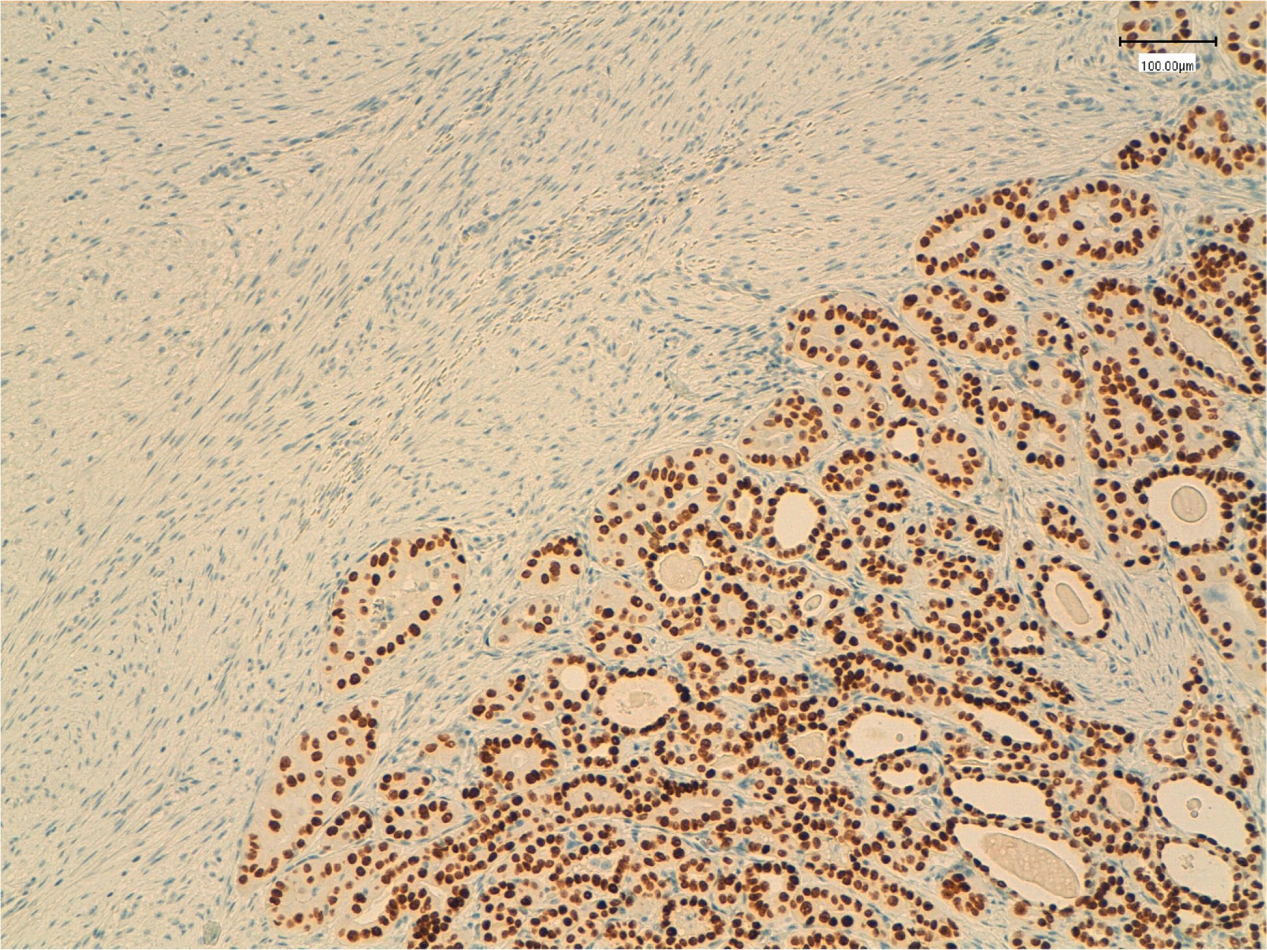
Immunohistochemical staining for PAX8 showing strong nuclear positivity in the epithelial component (right), while the stromal component is negative (left) (original magnification 100x)

**Fig. 5:**
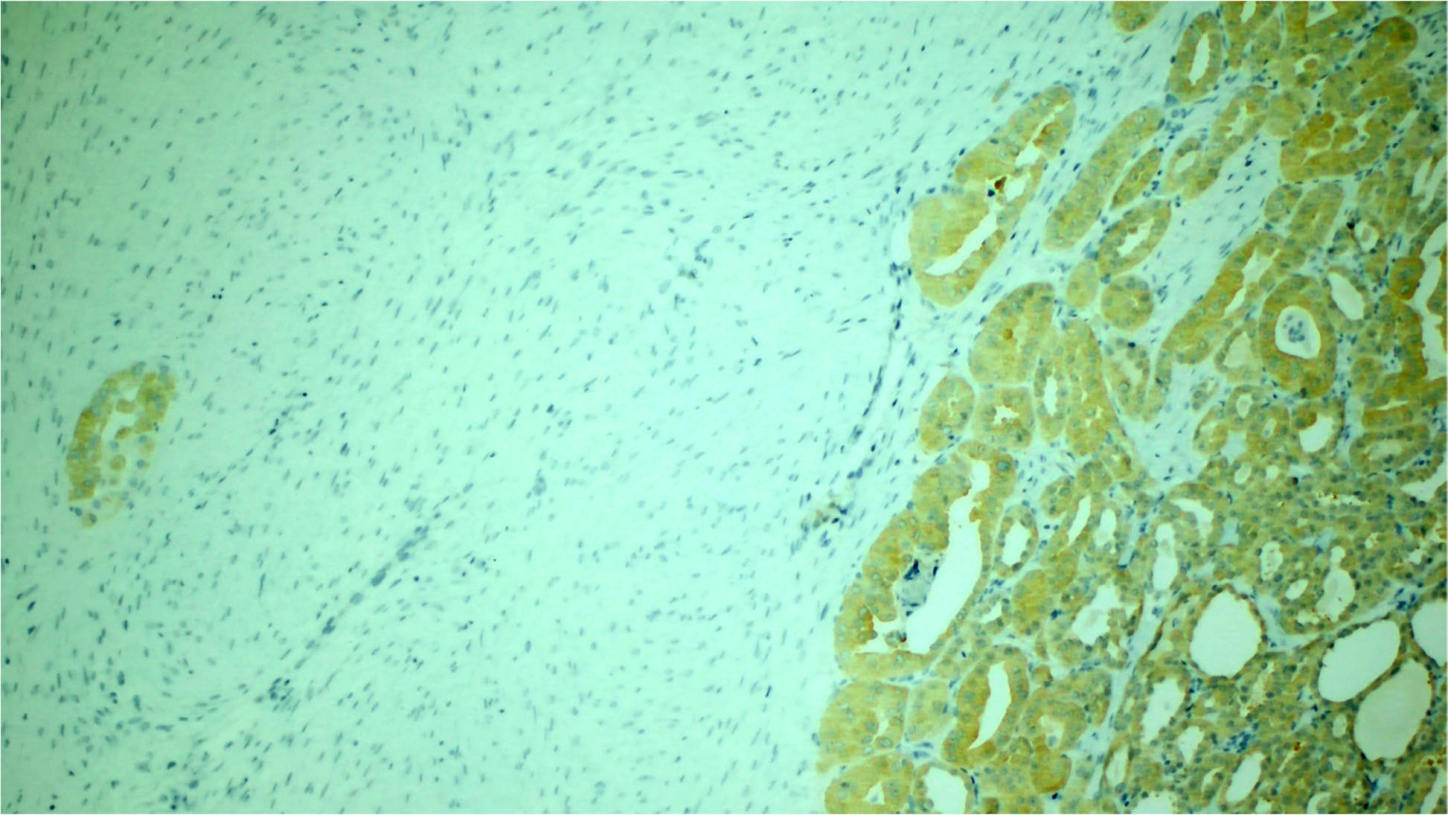
Immunohistochemical staining for BRAF V600E showing strong nuclera positivity in the epithelial component (right), while the stromal component is negative (left) ( magnification 5x)

Immunohistochemical analysis was performed on routinely processed, formalin-fixed, paraffin-embedded tissue sections. The epithelial cells were positive for PAX8, pancytokeratin (AE1/AE3), TTF-1 and membranous β-catenin. The stromal cell component was stained positively for smooth muscle actin (SMA) and focally for desmin and for β-catenin both in the nuclei and cytoplasm (Fig. 2) and negatively for PAX-8 (Fig. 3), TTF-1 (Fig. 4) and pancytokeratin (AE1/AE3). Additional immunohistochemical stain demonstrated a BRAF V600E mutation only in the epithelial component, while CTNNB1 mutation was found in the fibromatosis like stroma. (Fig. 5).

The results of the immunohistochemical stains are summarized in Table 1 and compared with other similar cases.

**Table 1:** Immunoreactivity of mesenchymal and papillary thyroid carcinoma cells

	Mesenchymal cells			Papillary thyroid carcinoma cells		
	Our case	Rebecchini et al (2 cases)	Ginter et al	Our case	Rebecchini et al (2 cases)	Ginter et al
β-Catenin	+ ^a^	+ ^a^	+ ^a^	+ ^c^	+ ^c^	n/a
SMA	+	+	+	-	-	-
Desmin	-/ + ^b^	-	-/ + ^b^	-	-	-
TTF1	-	-	-	+	+	+
AE1/AE3	-	-	-	+	+	+
PAX8	-	n/a	-	+	n/a	+
BRAF V600E	-	-	-	+	+	+
CTNNB1	+	+	n/a	-	-	n/a

^a^Cytoplasmic and nuclear staining; ^b^Focal stain; ^c^Membranous [3, 6]

## Discussion and conclusions

PTC-NFS and PTC-FMS are rare variants of papillary thyroid carcinoma. Papillary thyroid carcinoma with fibromatosis-like stroma was first described by Ostrowski et al. [7] in 1989, while papillary thyroid carcinoma with nodular fasciitis-like stroma was reported for the first time in 1991 by Chan et al. [8]. Since then, less than 60 cases have been described for both nosological definitions [4, 9]. The reported incidence of all these variants together is 0.5% of all papillary thyroid carcinoma in the series by Chan et al. [8], 0.17% in the series by Mizukami et al. [10] and 0.03% in the series by Rebecchini et al. [4]. It is not yet clear what this difference is due to, but some authors suggested that it could be related to different ethnic backgrounds [4, 8]. Like many other endocrine disorders, these types of tumors seem to be more prevalent in women, with a female-to-male ratio of 2.2:1 [9].

The histogenesis of PTC-NFS and PTC-FMS remains uncertain, but studies have demonstrated that one possible mechanism is an exaggerated mesenchymal reaction to injuries that results in the abundance of the stromal component. Since most patients underwent fine-needle aspiration (FNA) of the thyroid, some authors propose that these types of lesions are of an iatrogenic nature. This eventuality cannot be totally excluded, but it is unlikely to be true for the following reasons: these types of lesions can also be found in patients who were not subjected to FNA; there is no time correlation between FNA, morphologic changes, and surgery; the tumor’s stromal component is too diffused throughout the neoplasia to be reconducted to the focal stromal response to the FNA procedure [8]. Another hypothesis suggests a hormone dependent tumorigenesis nature, as estrogen receptors are involved in about one-third of PTC-FMS cases [10, 11]. Nevertheless, further investigation is needed.

In some reports, the terms PTC-NFS and PTC-FMS were used synonymously due to their morphological similarity. However, the distinction between nodular fasciitis and fibromatosis has important clinical implications: nodular fasciitis can be considered to be a self-limited and spontaneously regressing neoplasia, while fibromatosis has a more aggressive clinical course with a tendency for local recurrence [4]. The different behavior of these two variants is probably related to the aberrant β-catenin expression, which is common in desmoid-type fibromatosis and is closely associated with recurrence [4, 12].

In 2017, Rebecchini et al. performed a molecular analysis demonstrating that the epithelial and the mesenchymal component of the tumor present distinct somatic mutation: BRAF mutation in the epithelial component (V600E) and activating mutation of CTNNB1 in the mesenchymal component; thus, proposing the term “papillary thyroid carcinoma with desmoid type fibromatosis” (PTC-DTF). Similar results were obtained by Takada et al. [12], suggesting that an aberrant activation of the Wnt/β-catenin signaling is implicated in the tumor’s mesenchymal proliferation [4, 9, 12, 13]. Even if just few cases of PTC-FMS were reported to accurately assess the effect of specific mutation on prognosis and behavior of these tumors, mutation of CTNNB1 in the mesenchymal component is highly suggestive of PTC-FMS and thus aberrant β-catenin can be used as a diagnostic marker [13].

PTC-FMS cannot be differentiated from classical PTC based on ultrasonography [14] and the diagnosis can be difficult in both FNAC and histologic examination, but recognition is important to avoid diagnostic misinterpretation. The most important differential diagnosis to exclude is anaplastic carcinoma and carcinosarcoma, but usually the spindle cells of these entities show a greater degree of atypia compared to PTC-FMS. Other differential diagnosis includes fibroproliferative lesions of the thyroid, such as the fibrous variant of Hashimoto’s thyroiditis, post-operative spindle cells nodules and mesenchymal tumors. Recognition of the malignant epithelial component is essential in order to distinguish between these forms [6].

Given the rarity of PTC-FMS, there are no specific guidelines. PTC-FMS should be treated surgically like a low-grade malignant tumor, and a complete thyroidectomy needs to be performed to reduce the risk of recurrence [11]. In some cases, the procedure can be more difficult as the tumor can be locally more aggressive, invading the nerves and surrounding tissues. Nevertheless, with a radical approach the prognosis is similar to classical PTC.

The therapeutic process includes metabolic radiotherapy with I^131^ (RAI therapy) for the treatment of the malignant epithelial component, which was the only one identified in lymph node metastasis. On the other hand, RAI therapy appeared ineffective in the treatment of local recurrence, as the mesenchymal elements are not responsive to radioiodine [4]. Other systematic treatment options, used in extra-thyroidal desmoid tumors can be considered, including anti-hormonal therapy, non-steroidal anti-inflammatory drugs, tyrosine kinase inhibitors and chemotherapy [9].

Given the possibility of recurrence of PTC-FMS and the ineffectiveness of RAI therapy, complete surgical resection represents the main treatment for this type of tumor. Despite the fact that the specific nature of these lesions has yet to be determined, guidelines for classical PTC should be followed [4].

The awareness of this particular entity is important for a correct diagnosis and a more efficient management of patients with these types of tumors. As suggested by Roukain et al. [9], patients with advanced or progressive PTC-FMS tumors should be managed by multidisciplinary teams with experience in the management of thyroid carcinomas and sarcomas including endocrinologists, high-volume surgeons, oncologists, radiologists and nuclear medicine specialists.

## Data Availability

Not applicable.

## Abbreviations

PTC: Papillary Thyroid Carcinoma
PTC-FMS: Papillary Thyroid Carcinoma with Fibromatosis-like Stroma
PTC-NFS: Papillary Thyroid Carcinoma with Nodular Fasciitis-like Stroma
WHO: World Health Organization
TSH: Thyroid-Stimulating Hormone (TSH)
fT4: Free Thyroxine
fT3: Free Triiodothyronine
TPOAb: Thyroid Peroxidase Antibodies
TgAb: Thyroglobulin Antibodies
US: Ultrasonography
CT: Computed Tomography
FNAC: Fine-Needle Aspiration Cytology
ICCRTC: Italian Consensus for the Classification and Reporting of Thyroid Cytology
RAI: Radioactive Iodine
AJCC: America Joint Commission on Cancer
SMA: Smooth Muscle Actin
TTF-1: Thyroid Transcription Factor-1
FNA: Fine-Needle Aspiration
PTC-DTF: Papillary Thyroid Carcinoma with Desmoid-Type Fibromatosis
